# Longitudinal widefield OCT optic nerve thickness measurements in a case of incipient non-arteritic ischemic optic neuropathy

**DOI:** 10.1016/j.ajoc.2025.102367

**Published:** 2025-06-27

**Authors:** Winston Z. Lam, Mengxi Shen, Qinqin Zhang, Viet Hoan Le, Konica Singla, Marjan Imani Fooladi, Hong Jiang, Byron L. Lam, Ruikang K. Wang, Giovanni Gregori, Philip J. Rosenfeld

**Affiliations:** a Department of Ophthalmology, Bascom Palmer Eye Institute, University of Miami Miller School of Medicine, Miami, FL, USA; b Carl Zeiss Meditec, Inc., Dublin, CA, USA; c Department of Bioengineering, University of Washington, Seattle, WA, USA; d Department of Ophthalmology, University of Washington, Seattle, WA, USA; e Department of Neurology, University of Miami Miller School of Medicine, Miami, FL, USA

**Keywords:** Non-arteritic anterior ischemic optic neuropathy (NAION), Optic disc edema, Swept-source optical coherence tomography angiography (SS-OCTA), Incipient NAION, Age-related macular degeneration (AMD), Optic nerve head (ONH), Algorithm

## Abstract

**Purpose:**

Widefield swept-source optical coherence tomography angiography (SS-OCTA) scans were combined with a semi-automated algorithm for the early detection and resolution of optic nerve edema in a case of incipient non-arteritic anterior ischemic optic neuropathy (NAION).

**Observations:**

An incidental case of incipient NAION was identified in a 71-year-old woman enrolled in an ongoing prospective SS-OCTA imaging study using 12 × 12 mm scans that allowed imaging of both the macular and optic disc regions of patients with age-related macular degeneration (AMD). Early intervention with systemic corticosteroids led to the resolution of optic disc edema without subsequent vision loss. A novel semi-automated algorithm was used to quantify the onset and resolution of optic nerve edema and edema in the surrounding retina.

**Conclusions and importance:**

This use of 12 × 12 mm SS-OCTA scans and a semi-automated algorithm can significantly improve the detection and management of incipient NAION in clinical practice with the possibility that early detection would facilitate earlier intervention and better vision preservation in this condition. The use of widefield OCT imaging in conjunction with this novel algorithm in eyes at risk for optic nerve and macular edema could have broader implications for other retinal diseases in which optic disc and macular edema might arise such as diabetic retinopathy. Integrating this methodology into routine ophthalmic evaluations will enable clinicians to identify the onset of edema prior to vision loss, thereby improving patient outcomes.

## 1 Introduction

Non-arteritic anterior ischemic optic neuropathy (NAION) is characterized by ischemia in the optic nerve with oxygen deprivation causing damage to the retinal nerve fiber layer (RNFL) that results in optic nerve head (ONH) edema and may progress to cell death and associated vision loss.^1^, ^2^, ^3^, ^4^ Usually, vision loss occurs unilaterally and then stabilizes after a few weeks, but this vision loss is irreversible. Once NAION occurs in one eye, the fellow eye is known to have an increased risk for NAION in the future.^3^^,^^4^ NAION is more likely to occur in individuals who are older, of Caucasian descent, have existing vascular risk factors,^5^, ^6^, ^7^ and suffer from sleep apnea.^5^

While disc edema may resolve without additional complications, this outcome rarely happens. Usually, the disc edema progresses unnoticed until vision loss occurs. Several studies^1^, ^2^, ^3^, ^4^^,^^8^ found that asymptomatic disc edema is a precursor to a diagnosis of NAION, and this asymptomatic disc edema could be present for several weeks before vision loss due to NAION.

Incipient NAION is the diagnosis of exclusion in patients at risk for developing NAION and have asymptomatic disc edema.^3^, ^4^, ^5^, ^6^^,^^8^ In general, patients with asymptomatic ONH edema are older and have existing vascular risk factors or symptoms associated with NAION. Once NAION associated vision loss occurs, it's generally irreversible; however, early detection and treatment may improve the visual prognosis in these patients with incipient NAION.

Widefield swept-source optical coherence tomography angiography (SS-OCTA) is a non-invasive retinal imaging technique that provides an opportunity to detect incipient NAION before vision loss occurs. An advantage of using 12 × 12 mm SS-OCTA scans compared with the usual 6 × 6 mm macular scans is that the enlarged field-of-view (FOV) captures both the macula and optic nerve. The routine use of this larger 12 × 12 mm scan would be able to identify patients incidentally with incipient NAION while being followed for other age-related conditions such as age-related macular degeneration (AMD).

We present a case of an eye with intermediate AMD being followed in a natural history study that developed optic nerve edema detected on a 12 × 12 mm SS-OCTA scan without concomitant vision loss. A semi-automated algorithm for quantifying retinal and disc thickness changes over time was developed and used to quantify the disc edema before and after the use of systemic steroids.

## 2 Methods

In this retrospective review of eyes enrolled in a prospective natural history study of eyes with AMD, all eyes were scanned using SS-OCTA imaging (PLEX^Ⓡ^ Elite 9000, Carl Zeiss Medic, Inc. Dublin, CA) over a 12 × 12 mm FOV approximately every 1–3 months. A semi-automated algorithm was applied to measure retinal thickness and disc edema on serial 12 × 12 mm SS-OCTA scans. The 12 × 12 mm FOV covers both the macular area and the optic disc. Longitudinal follow up of these AMD patients allowed us to detect the onset and resolution of the disc edema. A novel algorithm was developed that used two segmentation boundaries between the inner limiting membrane (ILM) and retinal pigment epithelium (RPE) to compute thickness measurements. Manual adjustments were made to the segmentation lines as necessary, particularly at the optic nerve head, since the automatic segmentation of the RPE layer did not account for the break around the ONH. The graders were instructed to manually correct areas where the segmentation boundaries did not visually align with the ILM and RPE and in cases where the RPE was absent or not possible to follow, such as areas around the ONH, the RPE segmentation lines were manually drawn to connect the breaks around the ONH. These manual adjustments were made after reaching consensus grading by two graders (WL and MS) who were masked with regards to patient's visit. These results were then reviewed by a senior adjudicator (PJR). By using retinal OCTA en face images, longitudinal scans were registered to the scan when the disc edema was first detected. Registration among scans ensured that the same regions were compared longitudinally. The retinal and ONH thickness measurements were computed in regions of interest across the 12 × 12 mm FOV. In addition to the SS-OCTA scans, supplementary imaging included an optic disc cube scan to image the optic disc edema and retinal nerve fiber layer thickness, and a macular cube scan to measure the ganglion cell layer.

## 3 Case report

**Fig. 1:**
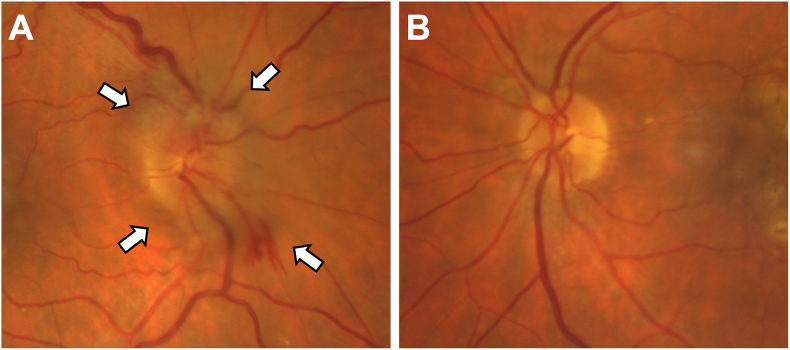
A**.** Fundus color image at the onset of right eye incipient NAION detected in November 2021 showing optic disc edema (white arrows). **B.** Left eye at time of presentation. (For interpretation of the references to color in this figure legend, the reader is referred to the Web version of this article.)

**Fig. 2:**
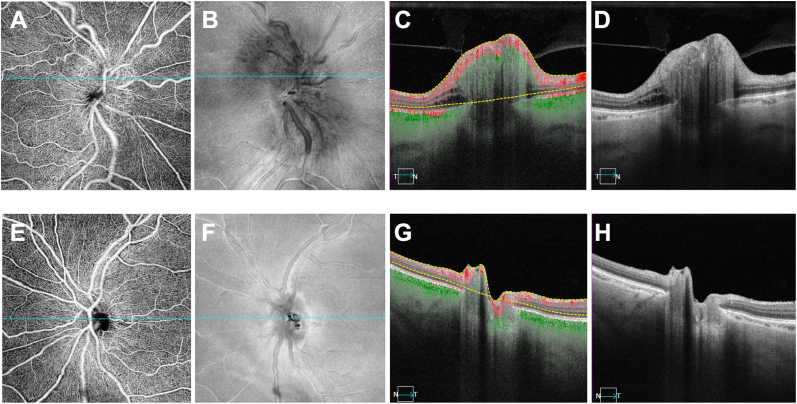
6 × 6 mm SS-OCTA images centered on the disc at time of incidental NAION right eye detection in November 2021.**A-D** Right eye. **A.** 6 × 6 mm SS-OCTA *en face* angiographic image of the disc. **B.** 6 × 6 mm SS-OCTA *en face* structural image of the disc. **C.** Angiographic B-scan taken of the disc edema with blood flow and segmentation lines. **D.** Structural B-scan of the disc edema. **E-H** Left eye. **E.** 6 × 6 mm SS-OCTA *en face* angiographic image of the disc. **F.** 6 × 6 mm OCTA *en face* structural image of the disc. **G.** Angiographic B-scan of the left eye across the disc with blood flow and segmentation lines. **H.** Structural B-scan of the disc.

A 71-year-old woman was diagnosed with incipient NAION at the time of a study visit to monitor dry AMD on 11/18/2021. The disc edema was found in her right eye as an incidental finding when imaging was performed at that study visit. This finding was first observed on a SS-OCTA 12 × 12 mm scan centered on the fovea and confirmed using color fundus imaging (Fig. 1) and 6 × 6 mm scans centered on the optic nerve (Fig. 2). No decreased capillary perfusion around the optic nerve was observed.

The patient had a history of hyperlipidemia, dry intermediate AMD, and obstructive sleep apnea, which was treated with a continuous positive airway pressure (CPAP) machine. No symptoms of visual acuity change or visual field defect were reported, and her best-corrected visual acuity (BCVA) was 20/30 over the course of follow-up with no visual loss on the Humphrey visual field (HVF) testing. She denied symptoms such as headaches, temple tenderness, jaw claudication, transient visual obscuration, diplopia, neck pain, and myalgias. Previous medical history included a recent right knee replacement and sciatica. The patient did not have hypertension, diabetes mellitus, or prior history of amiodarone usage, which may cause NAION.^5^^,^^6^

Two months before the ONH edema detection, 12 × 12 mm SS-OCTA scans were consistent with a diagnosis of dry AMD in both eyes. At the time of disc edema detection, a neuro-ophthalmologic work-up was conducted to exclude inflammatory, infectious, and neoplastic etiologies such as giant cell arteritis, sarcoidosis, vasculitis, syphilis, Bartonella, or Lyme disease. An orbital MRI with contrast was normal and laboratory testing unremarkable for abnormalities in her erythrocyte sedimentation rate (ESR), C-reactive protein (CRP), complete blood count (CBC), angiotensin-converting enzyme (ACE), antineutrophil cytoplasmic antibodies (ANCA), antinuclear antibody (ANA), rapid plasma reagin (RPR), fluorescent treponemal antibody absorption (FTA-ABS), Bartonella antibodies, and Lyme Western blot.

Given the negative work-up, she was diagnosed with incipient NAION. Risk factors for NAION included older age, hyperlipidemia, and a history of obstructive sleep apnea.^5^^,^^6^ The Humphrey visual field test did not show vision loss in the typical altitudinal patterns associated with classic NAION.^1^, ^2^, ^3^, ^4^^,^^8^ While the posterior hyaloid remained attached to the right optic nerve, traction was not apparent, and the degree of optic disc edema and hemorrhages were not typical for a vitreopapillary traction syndrome.^9^ The diagnosis of giant cell arteritis (GCA) was considered but ultimately excluded due to the absence of GCA symptoms, including jaw claudication, new-onset headaches, and temporal artery tenderness and laboratory test results were within normal ranges, which reduced the likelihood of any form of active systemic vasculitis including GCA. A biopsy was not performed due to the invasive nature of the procedure and the low likelihood of active systemic vasculitis.^10^

**Fig. 3:**
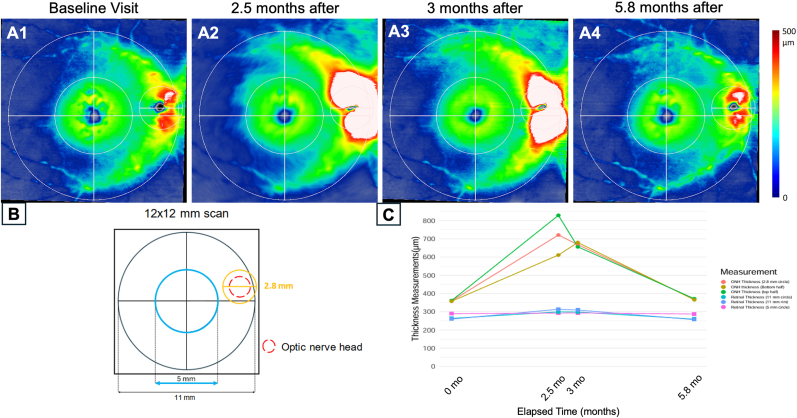
12 × 12 mm SS-OCTA thickness maps of the optic disc and macula with quantitation in the study eye using a novel algorithm **A1-A4** show right study eye thickness maps from a semi-automated algorithm over the course of 4 visits. **B.** Subregions within a 12 × 12 mm scan of a right eye where the thickness measurements were obtained. **C.** Change in thickness measurements from the subregions that included the optic nerve and retina over an x-axis proportional to time over the four visits. Note the absence of the disc edema in the first image, progression of disc edema in the second and third image, and resolution of disc edema in the last image.

**Fig. 4:**
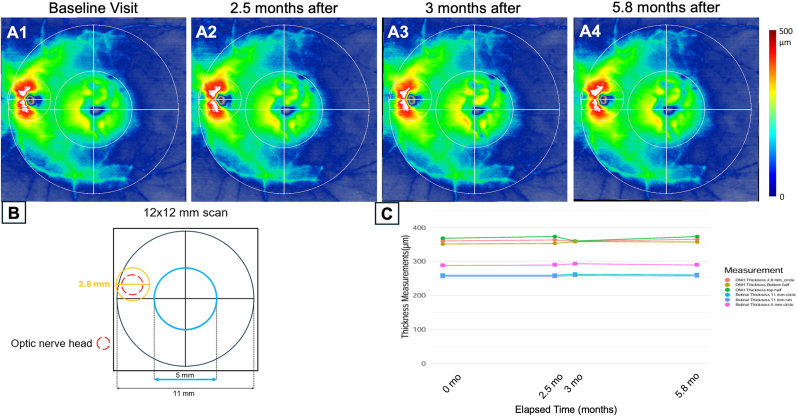
12 × 12 mm SS-OCTA thickness maps of the optic disc and macula with quantitation in the fellow eye using a novel algorithm **A1-A4** show the left fellow eye thickness maps from a semi-automated algorithm over the course of 4 visits. **B.** Subregions within a 12 × 12 mm scan of a left eye where the thickness measurements were obtained. **C.** Change in thickness measurements from the subregions that included the optic nerve and retina in the subregions over an x-axis proportional to time over the four visits. Note the absence of the disc edema in all the visits.

The patient was treated with prednisone 30 mg daily after the November 2021 visit for 2 weeks,^3^ then the prednisone was tapered to 20 mg daily and then tapered by 5 mg every 4 days until stoppage. Longitudinal thickness measurements of the optic nerve were obtained from a series of 12 × 12 mm SS-OCTA scans that included a visit prior to the onset of ONH edema, the visit when ONH edema was detected, and two follow up visits in December 2021 and February 2022 that showed subsequent resolution of the disc edema. In November 2021 (Fig. 3), the optic nerve thickness dramatically increased from the prior visit (Fig. 3A1), thicker superiorly than inferiorly. A small unmeasurable cup volume was observed on the optic disc cube 200 x 200 scan measurements. After prednisone administration, the optic nerve thickness measurements decreased by approximately 50 μm (Fig. 3C). Retinal thickness measurements were stable between November and December 2021. At the last visit over the course of the disease, the optic nerve edema resolved as the ONH thickness measurements returned to the baseline level from the prior visit in September 2021. Analysis of the fellow eye over the same period showed insignificant changes in thickness measurements of the optic nerve and retina (Fig. 4). The vision in the left eye remained at 20/30. After 3 years of follow up in November 2024, the optic nerve thickness and vision remained stable in both eyes.

## 4 Discussion

This case of a 71-year-old woman with intermediate AMD with asymptomatic unilateral disc edema diagnosed as incipient NAION showed resolution of her disc edema after prednisone therapy without vision loss, and 12 × 12 mm SS-OCTA scans were used to longitudinally monitor the onset and resolution of her clinical condition. Incipient NAION is rarely diagnosed due to the asymptomatic nature of the disease at this stage. While the true incidence of incipient NAION is unknown due to the lack of symptoms, research has shown that incipient NAION and optic disc edemas are a risk factor for the development of future NAION and subsequent involvement of the fellow eye. One study by Hayreh and Zimmerman in 2007^3^ found that of patients with asymptomatic disc edema, 55 % of cases had the fellow eye presenting with classic NAION and that 25 % of those with incipient NAION progressed to classic NAION in around 6 weeks. Interestingly, in 20 % of cases, classic NAION developed after the resolution of the initial episode of incipient NAION. Another case study by Subramanian et al.^4^ monitored 4 patients who developed vision loss attributable to NAION and found that all patients had either prior NAION or optic disc edema in the fellow eye.

We routinely monitor eyes in our retina clinic using both 6 × 6 mm and 12 × 12 mm scans centered on the fovea, and this case suggests that the added advantage of the 12 × 12 mm scan is the ability to identify the onset of incipient NAION, especially those with high-risk profiles. The use of the 12 × 12 mm scan allows for simultaneous monitoring of both the macula and optic nerve using a single scan pattern along with our semi-automated algorithm. The importance of this scan pattern is demonstrated by the asymptomatic nature of incipient NAION and the irreversible damage once NAION is diagnosed. In our case, we detected the disc edema during the patient's regular visit for AMD, allowing us to start steroid therapy. Had we not detected the optic disc edema and the presence of incipient NAION, the patient may have developed irreversible vision loss over the next few weeks or months if symptomatic NAION had developed, which still remains a long-term possibility. With a 12 × 12 mm scan and the new algorithm, we can detect any change in the disc region while monitoring AMD in the macula. The algorithm's ability to register and compare scans longitudinally has direct application in the clinical setting, aiding in the precise monitoring of disease progression or resolution. This capability is crucial for conditions like NAION, where changes in optic disc and retinal thickness can indicate the onset or advancement of the disease. This case suggests that if we detect disc edema early in the disease stage, we may be able to avoid progression to NAION and irreversible vision loss.

## 5 Conclusion

This study highlights the important role of advanced imaging techniques in the early detection and management of NAION, particularly in the asymptomatic stages that often precede significant visual loss. The use of a semi-automated algorithm to analyze 12 × 12 mm SS-OCTA scans has proven invaluable in this regard. The integration of widefield OCT imaging with algorithmic assistance holds great promise for the future of ocular disease management, offering a new paradigm in the early detection and treatment of conditions that could lead to irreversible visual impairment.

## CRediT authorship contribution statement

**Winston Z. Lam:** Writing – review & editing, Writing – original draft, Investigation, Formal analysis. **Mengxi Shen:** Writing – review & editing, Writing – original draft, Software, Methodology, Investigation, Formal analysis. **Qinqin Zhang:** Writing – review & editing, Software, Methodology, Formal analysis, Conceptualization. **Viet Hoan Le:** Writing – review & editing, Methodology, Investigation. **Konica Singla:** Writing – review & editing, Investigation. **Marjan Imani Fooladi:** Writing – review & editing, Investigation. **Hong Jiang:** Writing – review & editing, Investigation. **Byron L. Lam:** Writing – review & editing, Investigation. **Ruikang K. Wang:** Writing – review & editing, Investigation. **Giovanni Gregori:** Writing – review & editing, Investigation. **Philip J. Rosenfeld:** Writing – review & editing, Writing – original draft, Supervision, Project administration, Methodology, Investigation, Conceptualization.

## Patient Consent

Consent to publish this case report has been obtained from the patient(s) in writing.

## Acknowledgments and disclosures

a **Funding/Support:** Research supported by grants from 10.13039/501100002806Carl Zeiss Meditec, Inc., 10.13039/100017024the Salah Foundation, an unrestricted grant from the 10.13039/100010264Research to Prevent Blindness, Inc. (New York, NY), and the 10.13039/100000053National Eye Institute Center Core Grant (P30EY014801) to the Department of Ophthalmology, 10.13039/100006686University of Miami 10.13039/100008530Miller School of Medicine. The funding organizations had no role in the design or conduct of the present research.
b. **Financial Disclosures:** Giovanni Gregori and Philip J. Rosenfeld received research support from Carl Zeiss Meditec, Inc. Giovanni Gregori and the University of Miami co-own a patent that is licensed to Carl Zeiss Meditec, Inc. Dr. Rosenfeld also received research funding from Gyroscope/Novartis Therapeutics and Stealth BioTherapeutics. He is also a consultant for Apellis, Bayer Pharmaceuticals, Boehringer-Ingelheim, Carl Zeiss Meditec, Genentech/Roche, InflammX Therapeutics, Ocudyne, Regeneron Pharmaceuticals, and Unity Biotechnology. He also has equity interest in Apellis, CharacterBio, InflammX, Ocudyne, and Valitor. Ruikang K. Wang received financial support from Colgate Palmolive Company, Estee Lauder lnc., and is a consultant for Carl Zeiss Meditec and Cyberdontics. He also has several patents: US8, 750, 586, US8, 180, 134, US9, 282,905, US9, 759,544, US10, 354,378, US10, 529,061. The remaining authors have no disclosures.
c. **Authorship:** All authors attest that they meet the current ICMJE criteria for Authorship.

## Declaration of competing interest

**a. Funding/Support:** Research supported by grants from Carl Zeiss Meditec, Inc., the Salah Foundation, an unrestricted grant from the Research to Prevent Blindness, Inc. (New York, NY), and the National Eye Institute Center Core Grant (P30EY014801) to the Department of Ophthalmology, University of Miami Miller School of Medicine. The funding organizations had no role in the design or conduct of the present research.

**b. Financial Disclosures:** Giovanni Gregori and Philip J. Rosenfeld received research support from Carl Zeiss Meditec, Inc. Giovanni Gregori and the University of Miami co-own a patent that is licensed to Carl Zeiss Meditec, Inc. Dr. Rosenfeld also received research funding from Gyroscope/Novartis Therapeutics and Stealth BioTherapeutics. He is also a consultant for Apellis, Bayer Pharmaceuticals, Boehringer-Ingelheim, Carl Zeiss Meditec, Genentech/Roche, InflammX Therapeutics, Ocudyne, Regeneron Pharmaceuticals, and Unity Biotechnology. He also has equity interest in Apellis, CharacterBio, InflammX, Ocudyne, and Valitor. Ruikang K. Wang received financial support from Colgate Palmolive Company, Estee Lauder lnc., and is a consultant for Carl Zeiss Meditec and Cyberdontics. He also has several patents: US8, 750, 586, US8, 180, 134, US9, 282,905, US9, 759,544, US10, 354,378, US10, 529,061. The remaining authors have no disclosures.

☒ The authors declare the following financial interests/personal relationships which may be considered as potential competing interests.
